# Fluorescent nanodiamonds enable quantitative tracking of human mesenchymal stem cells in miniature pigs

**DOI:** 10.1038/srep45607

**Published:** 2017-03-30

**Authors:** Long-Jyun Su, Meng-Shiue Wu, Yuen Yung Hui, Be-Ming Chang, Lei Pan, Pei-Chen Hsu, Yit-Tsong Chen, Hong-Nerng Ho, Yen-Hua Huang, Thai-Yen Ling, Hsao-Hsun Hsu, Huan-Cheng Chang

**Affiliations:** 1 Institute of Atomic and Molecular Sciences, Academia Sinica, Taipei 106, Taiwan; 2 Department of Chemistry, National Taiwan University, Taipei 106, Taiwan; 3 Department of Pharmacology, College of Medicine, National Taiwan University, Taipei 100, Taiwan; 4 Department of Surgery, College of Medicine and the Hospital, National Taiwan University, Taipei 100, Taiwan; 5 Department of Obstetrics and Gynecology, College of Medicine and the Hospital, National Taiwan University, Taipei 100, Taiwan; 6 Department of Biochemistry and Molecular Cell Biology, School of Medicine, Graduate Institute of Medical Sciences, College of Medicine, Centre for Cell Therapy and Regeneration Medicine, and International PhD Program for Cell Therapy and Regeneration Medicine, Taipei Medical University, Taipei 110, Taiwan; 7 Department of Chemical Engineering, National Taiwan University of Science and Technology, Taipei 106, Taiwan

## Abstract

Cell therapy is a promising strategy for the treatment of human diseases. While the first use of cells for therapeutic purposes can be traced to the 19th century, there has been a lack of general and reliable methods to study the biodistribution and associated pharmacokinetics of transplanted cells in various animal models for preclinical evaluation. Here, we present a new platform using albumin-conjugated fluorescent nanodiamonds (FNDs) as biocompatible and photostable labels for quantitative tracking of human placenta choriodecidual membrane-derived mesenchymal stem cells (pcMSCs) in miniature pigs by magnetic modulation. With this background-free detection technique and time-gated fluorescence imaging, we have been able to precisely determine the numbers as well as positions of the transplanted FND-labeled pcMSCs in organs and tissues of the miniature pigs after intravenous administration. The method is applicable to single-cell imaging and quantitative tracking of human stem/progenitor cells in rodents and other animal models as well.

Mesenchymal stem cells (MSCs) are defined as self-renewing, multipotent progenitor cells with the capacity to differentiate into distinct mesenchymal lineages such as osteocytes, chondrocytes, and adipocytes^1^. Human MSCs are mainly found in bone marrow, adipose, and placenta tissues. These cells are one of the most promising sources of cell therapy and regenerative medicine for their multilineage differentiation potential and unique immunomodulatory properties^2^. They have been applied to treat various human diseases including cardiovascular disorder, lung fibrosis, liver diseases, and graft versus host diseases following bone marrow transplantation^3^,^4^. In light of the tremendous potential of this therapeutic approach, there is an imperative need to develop general and reliable methods to measure the biodistribution and pharmacokinetics of these cells *in vivo* for preclinical evaluation^5^. Such information is essential in clinical trials because it is vitally important to know whether the transplanted MSCs completely home to the target organs or they have unwanted homing that will induce inappropriate differentiation leading to cancer development^6^.

A number of attempts have previously been made to track human MSCs in murine xenogeneic models by using either polymerase chain reaction (PCR) to detect human DNA or immunostaining to identify human-specific nuclear proteins^7^,^8^. However, the data produced by these two methods provide little biodistribution information and are not quantitative enough to assess the safety and efficacy of these cells *in vivo*. In particular, when applied to large animals such as pigs, which share many physiological similarities with humans^9^, the PCR methods often yield unreliable results (Supplementary Table S1). More advanced methodologies (such as nanotechnologies^10^) are in demand.

An ideal cell tracking method should be biocompatible and nontoxic, require no genetic modification, have single-cell detection sensitivity, and permit quantification of cell numbers at any anatomic location^11^. Although X-ray, magnetic resonance, positron emission, gamma emission, and ultrasound-based modalities have enabled non-invasive imaging of transplanted stem cells *in vivo*^12^, quantifying cell numbers with these methods is difficult. A notable example is that of Templin *et al*.^13^ who used single photon emission computed tomography (SPECT) to track human-induced pluripotent stem cells after ^123^I or ^99m^Tc administration in a pig model. The method relied on radioisotope labeling and also the expression of transgenic sodium iodide symporters. It is technically demanding and only semi-quantitative information on the biodistribution of the transplanted cells could be obtained.

Optical imaging utilizing organic dyes, fluorescent proteins, or nanoparticles as exogenous contrast agents is more suitable for this purpose, although the technique is mainly used for small animal models in preclinical experimentation due to limited penetration depth of visible photons into tissue^14^,^15^. Among various exogenous contrast agents, fluorescent nanodiamond (FND) has recently emerged as an attractive option because the nanomaterial is chemically inert and inherently biocompatible^16^. Additionally, the particle contains a high-density ensemble of negatively charged nitrogen-vacancy (NV^−^) centers as built-in fluorophores, which are red fluorescent^17^, perfectly photostable^18^, and can be detected with high sensitivity in cells and tissues by fluorescence time gating^19^, microwave modulation^20^,^21^, and magnetic modulation^22^. These distinctive characteristics together make it possible to follow the biodistribution of human MSCs after FND labeling and subsequent transplantation in animals such as miniature pigs.

Figure 1 exhibits a schematic diagram of the experimental workflow. We used human MSCs isolated from placenta choriodecidual membrane in this study for their high availability and low ethical issues^23^,^24^. The cells, denoted as pcMSCs, were first labeled with albumin-conjugated FNDs and then transplanted into miniature pigs by intravenous administration. After sacrificing the animals at specific time points, we extracted and quantified the FNDs in five major organs by acid digestion and fluorescence intensity measurement. To minimize the sample loss, we applied a magnetic modulation technique to achieve background-free detection of FNDs directly in the digests without purification. Engraftment of the stem cells in tissues was finally identified by time-gated fluorescence imaging with single-cell resolution^19^.

## Results

### Quantification of FNDs

**Figure 2:**
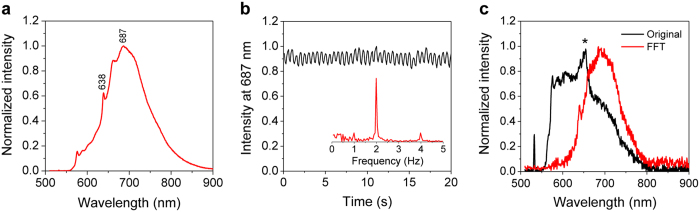
Detection of FNDs in aqueous solution by MMF. (**a**) Normalized fluorescence spectrum of FNDs in water (1 mg/mL), excited with a 532 nm laser. (**b**) Time trace of the peak fluorescence intensity at 687 nm under magnetic modulation of *f* = 2 Hz. Inset shows the corresponding frequency spectrum after FFT. (**c**) Normalized fluorescence spectrum of FNDs (5 μg/mL) in an acid digest of pig liver tissue (black) and its FFT spectrum (red) after demodulation at *f* = 2 Hz. The asterisk denotes the Raman peaks of water.

Taking advantage of the unique magneto-optical property of NV^−^ centers^25^, we first developed magnetically modulated fluorescence (MMF) into a background-free detection method to quantify FNDs in aqueous solution. The development is important because it allows direct quantification of FNDs in cells and tissue digests without pre-separation to avoid sample loss. The key instrument used in this quantification is a home-built MMF spectrometer (Supplementary Fig. S1). Figure 2a displays a typical fluorescence spectrum of 100-nm FNDs suspended in water (1 mg/mL) and excited by a 532 nm laser equipped in this spectrometer. The fluorescence intensity maximizes at 687 nm, corresponding to the phonon sidebands of an electronic transition of NV^−^ centers. When exposed to a time-varying magnetic field with a strength of *B* = 20 mT and switching on and off at a frequency of *f* = 2 Hz, the particles showed a modulation depth of ~5% in the fluorescence intensity (Fig. 2b). Fast Fourier transform (FFT) of the time trace yielded a distinct peak centered at 2 Hz (Fig. 2b). The peak height corresponds to the demodulated fluorescence intensity.

To prove the utility of the MMF method, we spiked FNDs (25 μg) in a sample solution (5 mL) prepared by digestion of pig liver tissue (1 g) in a concentrated mixture of HNO_3_ and H_2_O_2_ at 140 °C for several hours. As shown in Fig. 2c, the fluorescence spectrum of the solution was overwhelmed by the fluorescence and Raman signals of the digests. However, these signals exhibited no modulation when the sample solution was exposed to the time-varying magnetic field of *B* = 20 mT and *f* = 2 Hz. This characteristic allowed us to eliminate these background signals by FFT of the time traces of all wavelengths using a MATLAB program developed in-house for demodulation. The reduction of the background level was ~2 orders of magnitude, which enabled the recovery of the entire fluorescence spectrum of FNDs (5 μg/mL) in such a noisy environment with good signal-to-noise ratios (Fig. 2c).

**Figure 3:**
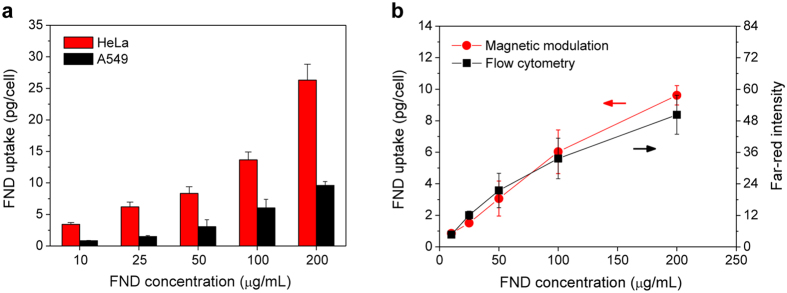
Quantification of FNDs in cells. (**a**) Dose-dependent uptakes of HSA-coated FNDs by A549 cells and HeLa cells in culture, analyzed by MMF. (**b**) Comparison of the uptakes of HSA-coated FNDs by A549 cells, analyzed by MMF and flow cytometry. Experiments were repeated in triplicate and error bars represent one standard deviation of uncertainty.

We next applied the background-free detection method to quantify the amount of FNDs taken up by cells. In this experiment, we first coated 100-nm FNDs with human serum albumin (HSA) by physical adsorption^26^ and then fed them to human lung adenocarcinoma epithelial cells (A549) or cervical cancer cells (HeLa) at the particle concentrations of 10–200 μg/mL. The cellular uptake was subsequently analyzed by MMF after removal of untaken HSA-FNDs in the medium. To conduct the analysis, a standard calibration curve was prepared by plotting the measured fluorescence intensity against the FND concentration gradient. The amounts of internalized FNDs were then determined from the fluorescence intensities of the HSA-FND-labeled cells (1 × 10^6^ cells/mL) in a cuvette (Supplementary Fig. S1) after sonication of these cells in water for 1 h to break up their plasma membrane in a glass test tube. For A549 cells incubated with 100 μg/mL HSA-FND at 37 °C for 4 h, we determined an average weight of 6.0 pg (or ~3.2 × 10^3^ particles) per cell for the internalized FNDs. The weight decreased monotonically with the decreasing HSA-FND concentration to zero (Fig. 3a). The result is in good correlation with flow cytometric analysis of the same cells incubated under the same conditions and detected in the far-red channel (Fig. 3b)^19^. It is known that flow cytometry provides only a relative measure for the degree of cell labeling. In contrast, MMF can reveal the absolute amounts of the labeling agents in the cells. Compared with A549, HeLa cells can take up twice more particles (Fig. 3a), reflecting a distinctly different endocytic behavior between these two types of human cell lines.

### Characterization of pcMSCs and FND-labeled pcMSCs

**Figure 4:**
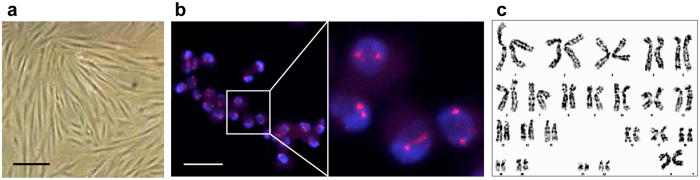
Characterization of pcMSCs. (**a**) pcMSCs in serum-free culture, displaying spindle-shaped morphology. Scale bar: 100 μm. (**b**) FISH analysis of stem cells isolated from the placentas of male newborns. X chromosomes are in red and cell nuclei in blue. The enlarged view shows two X chromosomes in the nucleus of each cell. Scale bar: 50 μm. (**c**) Karyotypical chromosome analysis of pcMSCs (*n* = 3). Only X chromosomes are detected in all three pcMSC samples.

The pcMSCs used in this study were isolated from the choriodecidual membrane of human placentas donated by women who had undergone cesarean sections. Uniquely, the cells could be cultured in serum-free defined medium and they displayed fibroblast-like morphology after attachment (Fig. 4a). More than 95% of the cell population expressed CD29, CD44, CD73, and CD90, and less than 2% of the population expressed CD14, CD34, CD45, and HLA-DR (Supplementary Fig. S2). *In vitro* assays for osteogenic, chondrogenic, and adipogenic differentiation of the cells all showed positive signals when stained with Alizarin Red S, Alcian Blue, and Oil Red O, respectively (Supplementary Fig. S3)^27^,^28^. Only XX chromosomes were detected by fluorescence *in situ* hybridization (FISH) (Fig. 4b and Supplementary Fig. S4). Further examination of the cells by karyotyping analysis found no evidence of Y chromosomes (Fig. 4c), confirming that the pcMSCs were derived from the maternal part (i.e. decidua basalis) of the placenta, irrespective of the gender of the newborns. No abnormal chromosomes were observed over 20 serial passages, proving the high stability of the cells under serum-free culture conditions.

**Figure 5:**
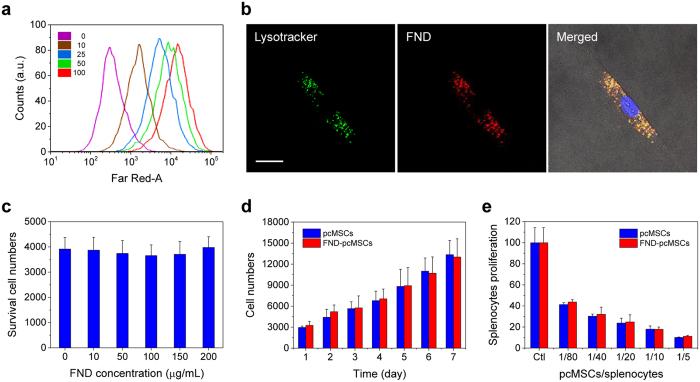
Characterization of HSA-FND-labeled pcMSCs. (**a**) Flow cytometric analysis of pcMSCs labeled with HSA-FNDs at the particle concentration of 0–100 μg/mL. (**b**) Confocal fluorescence images of pcMSCs labeled with Lysotracker and HSA-FND, and their merged image with the cell nucleus additionally stained in blue with Hoechst 33342. Scale bar: 20 μm. (**c**) Cell viability of pcMSCs labeled with HSA-FNDs at the particle concentration of 0–200 μg/mL. (**d**) Cell proliferation and (**e**) immunomodulation assays of pcMSCs labeled with HSA-FNDs at the particle concentration of 100 μg/mL. In the immunomodulation assays, unlabeled pcMSCs or FND-labeled pcMSCs were co-cultured with mouse splenocytes at the cell number ratios of 1:80 to 1:5. Ctl represents mouse splenocytes only. Experiments in (**c**–**e**) were repeated in triplicate and error bars represent one standard deviation of uncertainty.

In our first experiments with pcMSCs, the cells were grown and labeled by 100-nm FNDs coated with HSA. The purpose of the HSA coating is twofold: (1) to facilitate the dispersion of FNDs in cell medium during labeling and (2) to allow easy removal of free FNDs by phosphate-buffered saline washes after labeling. Similar to A549 and HeLa cells, the pcMSCs avidly took up the HSA-coated FND particles. For cells labeled at 100 μg/mL for 4 h, flow cytometric analysis indicated more than 98% of the pcMSCs containing HSA-FNDs (Fig. 5a). Colocalization studies showed that the internalized HSA-FNDs were predominantly trapped in lysosomes and did not enter cell nuclei (Fig. 5b). At 100 μg/mL, we determined an average weight of ~100 pg for the FNDs in one cell or an average number of ~5.5 × 10^4^ particles/cell by MMF (Supplementary Fig. S5). This amount is about 10 times as high as that taken up by A549 and HeLa cells (Fig. 3a). Remarkably, even with this large quantity of particles accumulated in the cytoplasm, the internalized HSA-FNDs do not seem to alter the cell viability (Fig. 5c) and proliferation (Fig. 5d) properties of the stem cells. No apparent exocytosis of FNDs was observed after 10 days of incubation in cell medium (Supplementary Fig. S6)^19^,^29^. Additionally, no significant difference between FND-labeled pcMSCs and untreated pcMSCs in the ability of suppressing the proliferation of mouse splenocytes was found by immunomodulation assays (Fig. 5e)^30^. All the results are ascribable to the exceptional inertness of the nanomaterial.

### Quantitative tracking of FND-labeled pcMSCs *in vivo*

**Figure 6:**
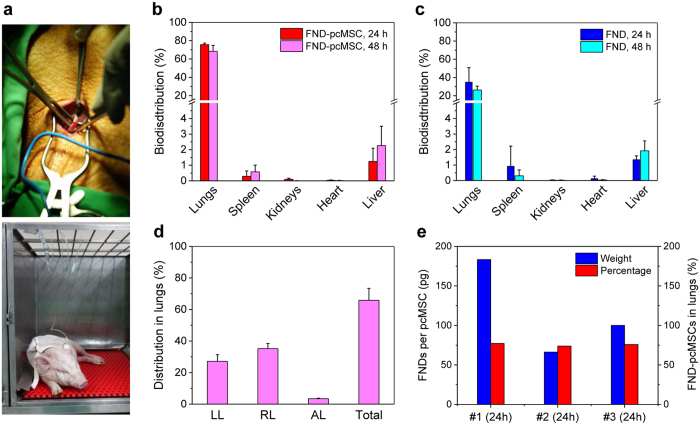
Quantitative tracking of HSA-FND-labeled pcMSCs in miniature pigs. (**a**) Central venous catheter insertion (top) and intravenous injection (bottom) of HSA-FND-labeled pcMSCs and HSA-FNDs into the pigs with a syringe pump. (**b**,**c**) Biodistribution of HSA-FND-labeled pcMSCs and HSA-FNDs in five different organs at two different time points. (**d**) Distribution of HSA-FND-labeled pcMSCs in different sections of the lungs, including left lobes (LL), right lobes (RL), and the accessory lobe (AL) at 48 h after injection. (**e**) Amounts of HSA-FNDs taken up by 3 sets of pcMSCs (blue) and the corresponding percentages of HSA-FND-labeled pcMSCs (red) found in the lungs 24 h after intravenous injection. The HSA-FND concentration used for the labeling was 100 μg/mL. Experiments in (**b**–**d**) were repeated in triplicate and error bars represent one standard deviation of uncertainty.

To conduct *in vivo* tracking, we injected HSA-FND-labeled pcMSCs into miniature pigs via their left internal jugular veins (Fig. 6a and Supplementary Fig. S7). A total of 12 miniature pigs were used and they were randomized into 4 groups. The pigs in each group received an injection of either HSA-FND-labeled pcMSCs (1 × 10^6^ cells/kg BW) or HSA-FNDs (0.1 mg/kg BW), which served as the control. After injection for 24 h or 48 h, the pigs were sacrificed and five major organs (including bilateral lungs, spleen, bilateral kidneys, heart, and liver) were collected for biodistribution measurement and fluorescence imaging. To enable FND quantification, we digested the organs in aqua regia/H_2_O_2_ mixtures to release the nanoparticles into the solution. Fluorescence intensities were then measured directly for FNDs in the tissue digests without extraction or other separation procedures to avoid loss of the particles during centrifugation or filtration treatment. Thanks to the chemical robustness of the nanomaterial, the unique magneto-optical properties of FNDs were still preserved and their fluorescence signals could be readily recovered from the acid digests by magnetic modulation at the concentration as low as 1 μg/mL. The recovery rate, determined by spiking a known amount of FNDs into the pig tissue followed by digestion through the same process, was more than 90%. Such quantitative analysis is inaccessible with molecular fluorophores such as organic dyes and fluorescent proteins due to the lack of chemical stabilities in strong acids.

A challenge in this experiment is that it is difficult to completely digest the whole organs collected from the sacrificed animals. Given a total weight of 20 kg for a miniature pig (Supplementary Table S2), organs such as the lungs have a typical wet weight of 200 g, which is about 1000 times greater than that of a mouse^31^,^32^. To overcome the challenge, in addition to using aqua regia/H_2_O_2_ mixtures, we employed acetone to dissolve lipids and a high-pressure autoclave reactor to facilitate the acid digestion of the whole organs. The combined approach has enabled us to determine the absolute amounts of FNDs (and thus the labeled cells) in the individual organs with both high sensitivity and accuracy. Figure 6b displays results of the experiment, where up to 70% of the HSA-FND-labeled pcMSCs were found to reside in the lungs of the miniature pigs after intravenous injection for 24–48 h. In contrast, the percentages of the HSA-coated FNDs in the same organs were only ~25% (Fig. 6c). Next to the lungs, the livers contained the highest amount (~3%) of FND-pcMSCs and also free HSA-coated FNDs. The contents of FNDs in the other three organs (spleen, kidneys, and heart) are all less than 1%. To the best of our knowledge, this is the first time that such quantitative information was obtained for stem cell tracking in a pig model.

The high sensitivity of this optical detection method, e.g. 1 μg FNDs out of 100 g organs or tissues, allows the determination of not only the biodistribution of pcMSCs in the animals but also their partition in different sections of organs or tissues. For the lungs, we found more HSA-FND-labeled pcMSCs residing in the right lobes than in the left lobes (Fig. 6d). Further analysis for the partition of the cells in six different lobe sections revealed that there were more pcMSCs retained in the upper and middle lobes than in the lower lobes of the lungs (Supplementary Fig. S8). It should be stressed that the presently observed biodistribution is independent of the amounts of FNDs taken up by the pcMSCs. Even with the amounts of HSA-FNDs in the cells varied by a factor of 2, the percentages of the pcMSCs detected in the lungs stayed nearly the same (Fig. 6e and Supplementary Fig. S9), which indicates the high reliability of this method.

**Figure 7:**
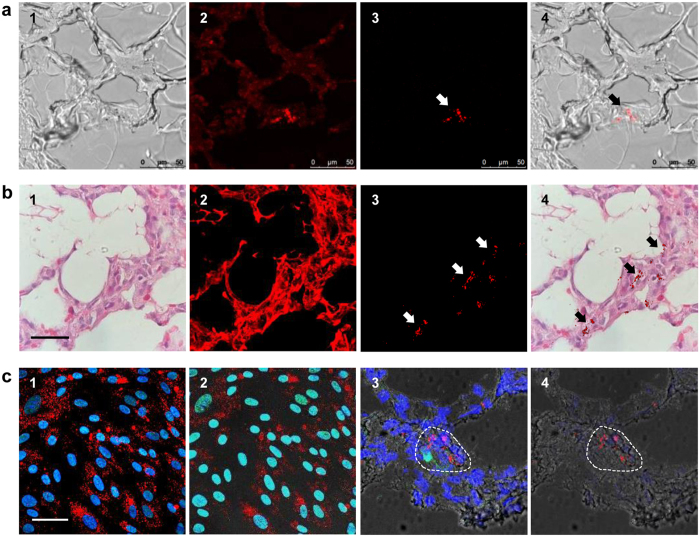
Fluorescence imaging of HSA-FND-labeled pcMSCs in pig tissues. (**a**) Fast screening of a lung tissue section before deparaffinization to find HSA-FND-labeled pcMSCs: (1) bright-field image, (2) fluorescence image without time gating, (3) fluorescence image with time gating at > 8 ns, and (4) merged images of (1) and (3). Scale bar: 50 μm. (**b**) Identification of HSA-FND-labeled pcMSCs in a lung tissue section by confocal microscopy: (1) H&E staining image, (2) confocal fluorescence image without time gating, (3) time-gated confocal fluorescence image, and (4) merged image of (1) and (3). White and black arrows denote HSA-FND-labeled pcMSCs. Scale bar: 20 μm. (**c**) Immunostaining of pcMSCs in culture and in a lung tissue section: (1) nuclei of cultured cells stained with Hoechst 33342 in blue and FNDs in red, (2) cells in (1) additionally stained with FITC-tagged antibodies for human-specific nuclei in green, (3) a lung tissue section stained with three fluorescent labels as in (2), and (4) photobleaching of the organic dyes (Hoechst 33342 and FITC) in (3) by repeated laser illumination of the specimen. Scale bar: 20 μm.

The large size of the pig organs does not prevent us from identifying the transplanted HSA-FND-labeled pcMSCs in their tissues. To accomplish this, we took advantage of another outstanding property of FND, that is, the NV^−^ centers in nanoscale diamonds have a fluorescence lifetime of ~20 ns, which is substantially longer than that (~3 ns) of cell autofluorescence^33^. We first performed fast screening of the tissue sections before deparaffinization by using fluorescence lifetime imaging microscopy (FLIM) or time-gated confocal fluorescence microscopy to achieve background-free detection (Fig. 7a and Supplementary Fig. S10). Subsequent imaging of FNDs in the deparaffinized samples with hematoxylin and eosin (H&E) staining allowed us to clearly identify the cells at the terminal bronchioles of the lungs (Fig. 7b). Further studies by immunostaining with FITC-tagged antibodies for human-specific nuclei confirmed that the two fluorescent labels (FITC and FND) are colocalized within the same cells (Fig. 7c). In contrast to organic dyes, which suffer from the problem of photobleaching, FND is non-photobleachable even under continuous, long-term high-power laser illumination. The easy identification of FNDs lends strong support for the validity and reliability of this nanotechnology-based method in finding transplanted human stem cells in the pig models.

## Discussion

In recent years, carbon nanomaterials (including carbon nanotube, graphene, and nanodiamond) have attracted considerable attention as promising fluorescent markers and drug delivery devices^34^. While some of the nanomaterials have found practical use in bioimaging and nanomedicinal therapy, absolute quantification of them in cells and tissues remains a challenge. This is mainly because it is difficult, if not impossible, to distinguish them from background carbon atoms by inductively coupled plasma mass spectrometry as typically conducted for metal, oxide, and semiconductor nanoparticles^35^. With the availability of FNDs, the quantification has become possible. In this work, we have developed MMF into an innovative method for absolute and sensitive quantification of FNDs in biological matrices. No extraction or other laborious separation steps prior to the fluorescence intensity measurement are required. Although magnetic modulation is less common than optical modulation, which has been successfully applied for probing photoswitchable fluorescent proteins^36^, it is more applicable to samples with strong light scattering and complex chemical compositions^37^. The method is ideally suitable for selective imaging of FNDs in tissues and also sensitive detection of FNDs in highly contaminated solution.

Lung failure is one of the leading causes of death worldwide. The lungs can be injured by trauma or affected by a number of diseases, including pneumonia and lung cancer. Recent discoveries of the therapeutic effects of human MSCs have made cell therapy an attractive option for lung injury treatment^38^,^39^. However, prior to the clinical trials, it is crucial to determine the ideal doses, injection speeds, biodistribution, physiological functions of the stem cells migrating to the target organs, and the lifelong duration of these cells before they are metabolized in host tissues. Such analysis will provide important pharmacokinetics, pharmacodynamics, and toxicology information for assessing the safety and efficacy of the MSC treatment. Although mice are the most commonly used animal models, the marked differences in size and structure of the peripheral airways between humans and rodents significantly limit their utility in preclinical settings.

We have chosen to use miniature pigs as the animal model in this study because they offer distinct advantages in both size and availability over non-human primates^9^. Additionally, their intact respiratory systems display human-like immune responses, similar to those of young adults^40^. Also, the anatomy and tissue structure of the airways and lungs of these animals resemble those of humans. With pcMSCs introduced into the miniature pigs by intravenous injection, our result shows that ~70% of the xenotransplanted cells are engrafted in the lungs for more than 48 h and these cells are predominantly located at the terminal bronchioles. It suggests a potential therapeutic use of pcMSCs to cure pulmonary disorders. Although the present study provides only the biodistribution and pharmacokinetics data of human stem cells in healthy pigs, the developed methods can be readily applied to diseased or injured animal models. In our future experiments, we will conduct investigations on lung-injured pigs and test the usefulness of the stem cells in repairing acute lung injury and treating other organ-specific diseases. Furthermore, efforts will be made to conjugate FNDs with superparamagnetic iron oxide nanoparticles to form dual-functional nanodevices for both magnetic resonance imaging and quantitative fluorescence tracking.

To conclude, we have demonstrated a viable application of FNDs for background-free imaging and quantitative tracking of human MSCs in animal models beyond rodents. The simplicity of this magneto-optical method, together with the chemical robustness and biological inertness of the nanomaterials as well as the large quantity of the nanoparticles taken up by the cells, has permitted studies of the biodistribution and pharmacokinetics of FND-labeled pcMSCs in miniature pigs for the first time. The technique is in excellent compatibility with FLIM and time-gated fluorescence imaging, which have been demonstrated to be a powerful means in acquiring high-contrast fluorescence images of FND-labeled cells in tissues. The ability to find single MSCs is particularly valuable for *ex vivo* histological detection of these cells in clinical trials. This combined approach represents an appealing alternative to hazardous radioisotope labeling techniques in cell tracking applications.

## Methods

### Chemicals

Cell media MCDB201, SMEM, DMEM, and RPMI 1640 were obtained from Life Technologies, human antibodies CD14-FITC, CD29-FITC, CD34-FITC, CD44-FITC, CD45-FITC, and CD90-FITC were from GeneTex, CD73-FITC and HLA-DR-PE were from eBioscience, mouse anti-human IgG1-FITC, IgG2a-FITC, and IgG2a-PE were from GeneTex, anti-CD3 and anti-CD28 were from BD Biosciences, Hoechst 33342, Lysotracker Green DND-265, and 6-carboxyfluorescein N-succinimidyl ester (CFSE) were from Invitrogen, Alizarin Red S, Alcian Blue, Oil Red O, phosphate-buffered saline (PBS), human serum albumin (HSA), erythrocyte lysis buffer, and all other chemicals were from Sigma-Aldrich and used without further purification.

### FND production

FNDs (~100 nm in diameter) were produced by radiation-damage of synthetic diamond powders (Micro + MDA M0.10, Element Six) with a 40-keV He^+^ beam, followed by vacuum annealing at 800 °C, air oxidation at 450 °C, and acid washes in concentrated H_2_SO_4_-HNO_3_ (3:1, v/v) at 100 °C, as previously described^41^,^42^.

### Protein conjugation

HSA conjugation was made by first sonicating the FND particles in distilled deionized water (DDW) for 15 min. They were then mixed with the protein at a weight ratio of FND:HSA ≈ 1:1 by gentle shaking at room temperature for 2 h to allow physical adsorption. After removal of unbound HSA by centrifugation, the precipitate was extensively washed with PBS.

### Cell culture and labelling

A549 or HeLa cells were seeded at a density of 2 × 10^5^ cells per 35-mm dish. They were maintained in regular Dulbecco’s modified Eagle’s medium (DMEM) supplemented with 10% fetal bovine serum (FBS) and new born calf serum at 37 °C with 5% CO_2_ in a humidified incubator overnight for attachment. Prior to cell labeling, HSA-coated FNDs were first dispersed in serum-free DMEM and then diluted with the medium to have a concentration in the range of 10–200 μg/mL. After adding the labeling medium (1 mL) to the dishes, cells were incubated at 37 °C for 4 h to facilitate uptake of the HSA-FND particles.

### Magnetically modulated fluorescence (MMF)

Fluorescence spectra of FNDs suspended in aqueous solution were acquired by using a MMF spectrometer built in house (Supplementary Fig. S1). The spectrometer was equipped with a continuous-wave 532 nm laser (DPGL-2100F, Photop Suwtech), a dichroic beam splitter (Z532RDC, Chroma), a long-working distance microscope objective (50×, NA 0.55, Mitutoyo), a long-pass edge filter (E550LP, Chroma), and a multichannel analyzer (C7473, Hamamatsu). Backward fluorescence was collected through the same objective to avoid spectral distortion due to strong light scattering by FNDs. To eliminate background noise, we magnetically modulated the FND fluorescence signal and then applied fast Fourier Transform (FFT) to extract concentration information from the measured fluorescence intensities. This was achieved by applying a time-varying magnetic field of 20 mT from a round electromagnet (EM400-12-212, APW) to the sample solution held in a 10 mm cuvette with a frequency of 2 Hz. For every wavelength, we obtained a time-domain spectrum with the magnetic field periodically switching on and off. A MATLAB program analyzed the time evolution of the spectra by performing FFT to yield intensities at the modulation frequency. The FND spectra were finally restored by plotting the demodulated fluorescence intensities against wavelength.

### pcMSC isolation

Human placentas were donated by women who had undergone cesarean sections with procedures approved by the local ethics committees of Taipei Medical University Hospital (TMU-JIRB 201501063). Written informed consent was obtained from all donors and experiments were performed in accordance with relevant guidelines and regulations. To isolate pcMSCs, choriodecidual membrane was first physically separated from the placentas and washed with Hank’s buffer to remove obvious blood clots. Clear choriodecidual tissues were then shredded with a surgical knife in a digestion buffer (SMEM medium supplemented with 0.5 mg/mL protease, 0.5 mg/mL collagenase B, and 1 mg/mL DNase I) and plated at 4 °C overnight. After pipetting and filtering the digestion buffer containing the tissue fractions through a 100-μm cell strainer, cells were collected by centrifugation and washed with blank medium several times. The cells were subsequently re-suspended in culture medium (MCDB201 supplemented with 1% insulin transferrin selenium, 10 ng/mL epidermal growth factor, and 1% penicillin/streptomycin) and planted in culture dishes coated with human collagen type IV. After 24 h of plantation, the dishes were shacked horizontally and washed with blank medium to remove non-adherent cells. Finally, the adherent cells were kept in the culture medium changed every 3–4 days.

### Immunophenotypic characterization

pcMSCs after several passages were characterized by flow cytometry with human antibodies against CD14-FITC, CD29-FITC, CD34-FITC, CD44-FITC, CD45-FITC, CD73-FITC, CD90-FITC, HLA-DR-PE, mouse anti-human IgG1-FITC, mouse anti-human IgG2a-FITC, and mouse anti-human IgG2a-PE for their immunophenotypes.

### Immunostaining and fluorescence *in situ* hybridization (FISH)

Immunostaining for human cell nuclei was carried out by using anti-nuclei antibodies (MAB1281, Merck Millipore) and FISH analysis by using the CEP X SpectrumOrange/Y SpectrumGreen Direct Labeled Fluorescent DNA Probe Kit (Abbott Molecular), both following the manufacturer’s protocols.

### *In vitro* differentiation assays

Three differentiation assays were performed as follows. (1) Osteogenesis: pcMSCs were plated at a density of 1 × 10^4^ cells/well in a 24-well plate and supplied with osteogenic induction medium (DMEM-high glucose with 10% FBS, 10 mM β-glycerosphate, 50 μg/mL ascorbic acid, and 0.1 μM dexamethasone). After 28 days of incubation, cells were fixed in 4% paraformaldehyde (PFA), washed with H_2_O, and stained with 40 mM Alizarin Red S. (2) Chondrogenesis: pcMSCs were plated at a density of 5 × 10^5^ cells/well in a 24-well plate and supplied with chondrogenic induction medium (DMEM-high glucose with 10% FBS, 10 ng/ml TGFβ-1, 1% ITS, and 50 μg/mL ascorbate). After 21 days of incubation, cells were fixed in 4% PFA, rinsed with 0.1 N HCl for 5 min, and stained with Alcian Blue. (3) Adipogenesis: pcMSCs were plated at a density of 6 × 10^4^ cells/well in a 24-well plate and supplied with adipogenic induction medium (DMEM-low glucose with 10% FBS, 1 μM dexamethasone, 0.5 mM 3-isobutyl-1-methylxanthine, 10 μg/mL insulin, and 100 μM indomethacin). After 21 days of incubation, cells were fixed and the lipid droplets were stained with Oil Red O using the mesenchymal adipogenesis kit (SCR020, Millipore).

### pcMSC labeling

pcMSCs after attachment were labeled with HSA-coated FNDs (10–200 μg/mL) through endocytosis at 37 °C with 5% CO_2_ for 4 h. The HSA-FND-labeled pcMSCs were then thoroughly washed with PBS to remove untaken FNDs. This was followed by trypsinization and analysis using a flow cytometer (FACSAria II, BD Bioscience) equipped with a 532 nm laser for the cellular uptake. To enable fluorescence imaging, the cells were additionally labeled with Hoechst 33342 and Lysotracker Green DND-26 for nuclei and lysosomes, respectively.

### Cell viability assay

A cell counting kit (CCK-8, Sigma) was used to evaluate the cell viability after FND labeling. First, pcMSCs (5 × 10^3^ cells) were plated in a 48-well plate for 24 h and then labeled with 10–200 μg/mL HSA-coated FND for 4 h. After PBS wash to remove untaken FNDs, WST-8 (a water-soluble tetrazolium salt) was added to the wells and incubated for 3 h. Absorbance was measured at 450 nm using a microplate reader (BioTek) and converted into cell numbers according to the calibration curve made with the known numbers of pcMSCs (1 × 10^3^–1 × 10^5^ cells/well) in the plate.

### Cell proliferation assay

pcMSCs were labeled with 100 μg/mL HSA-coated FND for 4 h. Next, the cells were trypsinized and replated at a density of 3 × 10^3^ cells/well in a 48-well plate. The numbers of live cells were measured by using the cell counting kit (CCK-8, Sigma) every day from day 1 to day 7.

### Immunomodulation assay

Spleens isolated from C57BL/6 mice were mechanically shredded in RPMI 1640 medium supplemented with 10% FBS, 1% L-Glutamine, 100 U/mL penicillin and 100 μg/mL streptomycin to release splenocytes. After standing for 10 min, the upper portion of the medium was collected and centrifuged to obtain the primary splenocytes in the pellets. This was followed by the addition of erythrocyte lysis buffer to the pellets for 1 min, stopping of the reaction with the RPMI 1640 medium, and isolation of the splenocytes by centrifugation. After an additional wash with the same medium to remove traces of the lysis buffer, the splenocytes were re-suspended in culture medium and incubated in a humidified chamber with 5% CO_2_/air mixture at 37 °C.

Immunomodulation assays were performed by labeling splenocytes with 5 μM CFSE at room temperature, following the manufacturer’s protocol. The CFSE-labeled splenocytes (1 × 10^6^ cells/well) were then co-cultured with either unlabeled pcMSCs or FND-labeled pcMSCs (1 × 10^4^–2 × 10^5^ cells/well) in a 24-well plate. The splenocyte proliferation rates were finally assessed by flow cytometry to determine the numbers of CFSE-positive cells at 48 h after activation with 0.1 μg/mL anti-CD3 and anti-CD28.

### Animal experiments

Miniature pigs (weighing 18–24 kg each) were obtained from the Experimental Farm of National Taiwan University or the Taitung Animal Propagation Station, Taiwan. They were pair-housed on a 12-h light/dark schedule (lights on at 0700) with controlled temperature and humidity. Food and water were available *ad libitum*. The study was approved by the Institutional Animal Care and Use Committee (with IACUC Approval No: 20140478) of College of Medicine and College of Public Health, National Taiwan University. All experiments were performed in accordance with the guidelines and regulations established in the Guide for the Care and Use of Laboratory Animals.

Twelve miniature pigs were used in this study. They were randomized into 4 groups, each consisting of 3 pigs. Prior to the experiments, the pigs were fasted overnight and premedicated intramuscularly with atropine (0.1 mg/kg BW), zoletil 50 (10 mg/kg BW), and xylazine (10 mg/kg BW) to allow placement of an intravenous catheter and continuous electrocardiogram and pulse oximetry monitoring. After mask preoxygenation, anesthesia was induced with propofol (2–3 mg/kg BW) and succinylcholine (1 mg/kg BW) by intravenous bolus via an ear vein, and the trachea was orally intubated with a 6.0–8.0 mm inner diameter endotracheal tube. The pigs were provided mechanical ventilation until breathing spontaneously following initial anesthesia at a volume-controlled setting of 20 mL/kg BW (body weight). During preparation, the respiratory frequency was adjusted at 15–20 breaths/min to maintain the mean end-tidal CO_2_ pressure at 35–40 mmHg and the inspiratory oxygen concentration was titrated to maintain saturation (>96%), measured by pulse oximetry. Titrated anesthesia was maintained by propofol infusion with a rate of 10 mg/kg/h, guided by the prospectively set target parameters for heart rate, blood pressure, tail and hoof pinch response, and spontaneous breathing during the entire course of the experiment.

A central venous catheter was placed in the left or right internal jugular vein of the pig being experimented on under sterile conditions. The animal was administered with a dose of 500 U heparin to prevent venous catheter clot formation and a dose of 1.5 million U penicillin to prevent infection. Each pig received an intravenous injection of HSA-FNDs (served as the control group) or HSA-FND-labeled pcMSCs (served as the treatment group) infused at a dose of 1 × 10^6^ cells/kg BW in 60 mL by using a syringe pump at a rate of 150 mL/h. After injection of the particles or cells for 24 h or 48 h, the pigs were sacrificed and five major organs (including bilateral lungs, spleen, bilateral kidneys, heart, and liver) were collected for fluorescence imaging and biodistribution measurements.

### Fluorescence imaging

Confocal fluorescence imaging was conducted by using an inverted confocal microscope (Leica, SP8) equipped with a white-light continuum laser operating at 405, 488, and 561 nm for the excitation of Hoechst 33342, Lysotracker Green DND-26 (or FITC), and FND, respectively. Fluorescence was collected through an oil-immersion objective (63 × , NA 1.4) and detected with either a photomultiplier (PMT) for Hoechst 33342 and Lysotracker Green DND-26 (or FITC) or a hybrid detector (HyD) for FND at emission wavelengths of 420–450 nm, 500–550 nm, and 700–800 nm, respectively. The use of HyD allowed effective removal of unwanted background signals by time gating.

### Biodistribution measurement

Organs collected from sacrificed pigs were weighted and dried in oven. After being cut into small pieces and mixed together, they were divided into several parts and each part (typically 20 g) was placed in a Florence flask (50 mL) containing aqua regia (24 mL) and 30% H_2_O_2_ (5 mL) heated to 85–100 °C for digestion until the solution became transparent. Indigestible portions were then collected by centrifugation and dissolved in acetone to remove lipid components. The remaining indigestible and undissolvable tissues were collected by centrifugation, placed in a Teflon jar containing 70% HNO_3_ (5 mL) in an autoclave reactor (BO-300, Hondwen), and heated to 90–200 °C under high pressure for 2 h. The residues were finally digested in 70% HNO_3_ at 140 °C in a microwave reactor (Discover BenchMate, CEM) for 1.5 h and then added with 30% H_2_O_2_ for decoloration. The reaction continued until the solution became clear. All fractions were finally pooled together for fluorescence intensity measurements.

## Additional Information

**How to cite this article:** Su, L.-J. *et al*. Fluorescent Nanodiamonds Enable Quantitative Tracking of Human Mesenchymal Stem Cells in Miniature Pigs. *Sci. Rep.* **7**, 45607; doi: 10.1038/srep45607 (2017).

**Publisher's note:** Springer Nature remains neutral with regard to jurisdictional claims in published maps and institutional affiliations.

## Supplementary Material

Supplementary Information

## Figures and Tables

**Figure 1:**
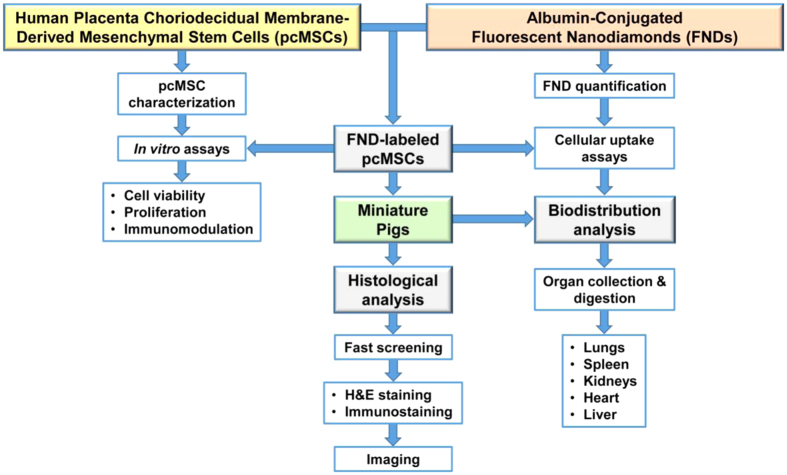
Schematic of the experimental workflow. Major procedures include pcMSC isolation, FND labeling, *in vitro* assays, intravenous injection of FND-labeled pcMSCs into miniature pigs, and quantification of FNDs extracted from organs of the xenotransplanted pigs.

## References

[b1] Dominici M. . Minimal criteria for defining multipotent mesenchymal stromal cells. The International Society for Cellular Therapy position statement. Cytotherapy 8, 315–317 (2006). 16923606 10.1080/14653240600855905

[b2] Parekkadan B. & Milwid J. M. Mesenchymal stem cells as therapeutics. Annu. Rev. Biomed. Eng. 12, 87–117 (2010). 20415588 10.1146/annurev-bioeng-070909-105309 PMC3759519

[b3] Quarto R. . Repair of large bone defects with the use of autologous bone marrow stromal cells. N. Engl. J. Med. 344, 385–386 (2001). 11195802 10.1056/NEJM200102013440516

[b4] Wang S., Qu X. & Zhao R. C. Clinical applications of mesenchymal stem cells. J. Hematol. Oncol. 5, 19 (2012). 22546280 10.1186/1756-8722-5-19 PMC3416655

[b5] Sensebé L. & Fleury-Cappellesso S. Biodistribution of mesenchymal stem/stromal cells in a preclinical setting. Stem Cells Int. 2013, 678063 (2013). 24222773 10.1155/2013/678063 PMC3810433

[b6] Breitbach M. . Potential risks of bone marrow cell transplantation into infarcted hearts. Blood 110, 1362–1369 (2007). 17483296 10.1182/blood-2006-12-063412

[b7] Meyerrose T. E. . *In vivo* distribution of human adipose-derived mesenchymal stem cells in novel xenotransplantation models. Stem Cells 25, 220–227 (2007). 16960135 10.1634/stemcells.2006-0243 PMC4382309

[b8] Toupet K. . Long-term detection of human adipose derived mesenchymal stem cells after intra-articular injection. Arthritis Rheumatism 65, 1786–1794 (2013). 23553439 10.1002/art.37960

[b9] Vodicka P. . The miniature pig as an animal model in biomedical research. Ann. N. Y. Acad. Sci. 1049, 161–171 (2005). 15965115 10.1196/annals.1334.015

[b10] Ferreira L., Karp J. M., Nobre L. & Langer R. New opportunities: the use of nanotechnologies to manipulate and track stem cells. Cell Stem Cell 3, 136–146 (2008). 18682237 10.1016/j.stem.2008.07.020

[b11] Frangioni J. V. & Hajjar R. J. *In vivo* tracking of stem cells for clinical trials in cardiovascular disease. Circulation 110, 3378–3383 (2004). 15557385 10.1161/01.CIR.0000149840.46523.FC

[b12] Kircher M. F., Gambhir S. S. & Grimm J. Noninvasive cell-tracking methods. Nat. Rev. Clin. Oncol. 8, 677–688 (2011). 21946842 10.1038/nrclinonc.2011.141

[b13] Templin C. . Transplantation and tracking of human-induced pluripotent stem cells in a pig model of myocardial infarction: assessment of cell survival, engraftment, and distribution by hybrid single photon emission computed tomography/computed tomography of sodium iodide symporter transgene expression. Circulation 126, 430–439 (2012). 22767659 10.1161/CIRCULATIONAHA.111.087684

[b14] Leblond F., Davis S. C., Valdes P. A. & Pogue B. W. Pre-clinical whole-body fluorescence imaging: Review of instruments, methods and applications. J. Photochem. & Photobiol. B 98, 77–94 (2010). 20031443 10.1016/j.jphotobiol.2009.11.007 PMC3678966

[b15] de Almeida P. E., van Rappard J. R. M. & Wu J. C. *In vivo* bioluminescence for tracking cell fate and function. Am. J. Physiol. Heart Circ. Physiol. 301, H663–H671 (2011). 21666118 10.1152/ajpheart.00337.2011 PMC3191083

[b16] Vaijayanthimala V., Tzeng Y.-K., Chang H.-C. & Li C.-L. The biocompatibility of fluorescent nanodiamonds and their mechanism of cellular uptake. Nanotechnology 20, 425103 (2009). 19779240 10.1088/0957-4484/20/42/425103

[b17] Yu S.-J., Kang M.-W., Chang H.-C., Chen K.-M. & Yu Y.-C. Bright fluorescent nanodiamonds: no photobleaching and low cytotoxicity. J. Am. Chem. Soc. 127, 17604–17605 (2005). 16351080 10.1021/ja0567081

[b18] Fu C.-C. . Characterization and application of single fluorescent nanodiamonds as cellular biomarkers. Proc. Natl. Acad. Sci. USA 104, 727–732 (2007). 17213326 10.1073/pnas.0605409104 PMC1783382

[b19] Wu T.-J. . Tracking the engraftment and regenerative capabilities of transplanted lung stem cells using fluorescent nanodiamonds. Nat. Nanotechnol. 8, 682–689 (2013). 23912062 10.1038/nnano.2013.147 PMC7097076

[b20] Igarashi R. . Real-time background-free selective imaging of fluorescent nanodiamonds *in vivo*. Nano Lett. 12, 5726–5732 (2012). 23066639 10.1021/nl302979d

[b21] Hegyi A. & Yablonovitch E. Molecular imaging by optically detected electron spin resonance of nitrogen-vacancies in nanodiamonds. Nano Lett. 13, 1173–1178 (2013). 23384363 10.1021/nl304570b

[b22] Sarkar S. K. . Wide-field *in vivo* background free imaging by selective magnetic modulation of nanodiamond fluorescence. Biomed. Opt. Express 5, 1190–1202 (2014). 24761300 10.1364/BOE.5.001190 PMC3985990

[b23] In’t Anker P. S. . Isolation of mesenchymal stem cells of fetal or maternal origin from human placenta. Stem Cells 22, 1338–1345 (2004). 15579651 10.1634/stemcells.2004-0058

[b24] Evangelista M., Soncini M. & Parolini O. Placenta-derived stem cells: new hope for cell therapy? Cytotechnology 58, 33–42 (2008). 19002775 10.1007/s10616-008-9162-z PMC2593758

[b25] Schirhagl R., Chang K., Loretz M. & Degen C. L. Nitrogen-vacancy centers in diamond: nanoscale sensors for physics and biology. Annu. Rev. Phys. Chem. 65, 83–105 (2014). 24274702 10.1146/annurev-physchem-040513-103659

[b26] Tzeng Y.-K. . Superresolution imaging of albumin-conjugated fluorescent nanodiamonds in cells by stimulated emission depletion. Angew. Chem. Int. Ed. 50, 2262–2265 (2011). 10.1002/anie.201007215 21351332

[b27] Gregory C. A., Gunn W. G., Peister A. & Prockop D. J. An Alizarin red-based assay of mineralization by adherent cells in culture: comparison with cetylpyridinium chloride extraction. Anal. Biochem. 329, 77–84 (2004). 15136169 10.1016/j.ab.2004.02.002

[b28] Guasti L. . High plasticity of pediatric adipose tissue-derived stem cells: too much for selective skeletogenic differentiation? Stem Cells Transl. Med. 1, 384–95 (2012). 23197817 10.5966/sctm.2012-0009 PMC3659709

[b29] Fang C.-Y. . The exocytosis of fluorescent nanodiamond and its use as a long-term cell tracker. Small 7, 3363–3370 (2011). 21997958 10.1002/smll.201101233

[b30] Glennie S., Soeiro I., Dyson P. J., Lam E. W. & Dazzi F. Bone marrow mesenchymal stem cells induce division arrest anergy of activated T cells. Blood 105, 2821–2827 (2005). 15591115 10.1182/blood-2004-09-3696

[b31] Yuan Y., Chen Y. W., Liu J.-H., Wang H. F. & Liu Y. F. Biodistribution and fate of nanodiamonds. Diamond Relat. Mater. 18, 95–100 (2009).

[b32] Vaijayanthimala V. . The long-term stability and biocompatibility of fluorescent nanodiamond as an *in vivo* contrast agent. Biomaterials 33, 7794–7802 (2012). 22863379 10.1016/j.biomaterials.2012.06.084

[b33] Billinton N. & Knight A. W. Seeing the wood through the trees: a review of techniques for distinguishing green fluorescent protein from endogenous autofluorescence. Anal. Biochem. 291, 175–197 (2001). 11401292 10.1006/abio.2000.5006

[b34] Hong G., Diao S., Antaris A. L. & Dai H. Carbon nanomaterials for biological imaging and nanomedicinal therapy. Chem. Rev. 115, 10816–10906 (2015). 25997028 10.1021/acs.chemrev.5b00008

[b35] Vanhecke D. . Quantification of nanoparticles at the single-cell level: an overview about state-of-the-art techniques and their limitations. Nanomedicine (Lond) 9, 1885–1900 (2014). 25325243 10.2217/nnm.14.108

[b36] Hsiang J.-C., Jablonski A. E. & Dickson R. M. Optically modulated fluorescence bioimaging: visualizing obscured fluorophores in high background. Acc. Chem. Res. 47, 1545–1554 (2014). 24725021 10.1021/ar400325y PMC4033652

[b37] Yang N. & Cohen A. E. Optical imaging through scattering media via magnetically modulated fluorescence. Opt. Express 18, 25461–25467 (2010). 21164893 10.1364/OE.18.025461

[b38] Yang J. & Jia Z. Cell-based therapy in lung regenerative medicine. Regen Med. Res. 2, 7 (2014). 25984335 10.1186/2050-490X-2-7 PMC4389643

[b39] Ho M. S., Mei S. H. & Stewart D. J. The Immunomodulatory and therapeutic effects of mesenchymal stromal cells for acute lung injury and sepsis. J. Cell Physiol. 230, 2606–2617 (2015). 25913273 10.1002/jcp.25028

[b40] Kirschvink N. & Reinhold P. Use of alternative animals as asthma models. Curr. Drug Targets 9, 470–484 (2008). 18537586 10.2174/138945008784533525

[b41] Chang Y.-R. . Mass production and dynamic imaging of fluorescent nanodiamonds. Nat. Nanotechnol. 3, 284–288 (2008). 18654525 10.1038/nnano.2008.99

[b42] Su L.-J. . Creation of high density ensembles of nitrogen-vacancy centers in nitrogen-rich type Ib nanodiamonds. Nanotechnology 24, 315702 (2013). 23857995 10.1088/0957-4484/24/31/315702

