# Supplementary material for: Fluorescent nanodiamonds enable quantitative tracking of human mesenchymal stem cells in miniature pigs
Source: Sci Rep. 2017 Mar 30;7:45607. doi: 10.1038/srep45607 (PMC5372358; doi:10.1038/srep45607)
Supplement: Supplementary Information [file srep45607-s1.pdf]

Supplementary Information

Fluorescent Nanodiamonds Enable Quantitative Tracking of Human Mesenchymal Stem Cells in Miniature Pigs

L.-J. Su, M.-S. Wu, Y. Y. Hui, B.-M. Chang, L. Pan, P.-C. Hsu, Y.-T. Chen, H.-N. Ho, Y.-H. Huang, T.-Y. Ling, H.-H. Hsu, and H.-C. Chang

–

DNA extraction and real-time PCR. Lung tissues (25 mg) from xenotransplanted pigs were homogenized in liquid nitrogen. They were treated with the Purelink Genomic DNA Mini kit (Invitrogen) to extract the genomic DNA of the tissues, following the manufacturer's protocol. Real-time PCR assays were performed in a thermal cycler (ABI 7300, Life Technologies), and each measurement consisted of 12.5 μL TaqMan Universal PCR Master Mix (Applied Biosystems) supplemented with the primer (Alu sequence), TaqMan probe, and DNA templates in a final volume of 25 μL. After incubation of the mixtures at 50 °C for 2 min and 95 ℃ for 10 min, the PCR reactions were carried out for 40 cycles at 95 ℃ for 15 s and 60 ℃ for 1 min. The control experiment consisted of pcMSCs containing a total amount of 200 ng human DNA, which served as the basis to calculate the total amounts of human DNA in the pig's lungs. The assays were repeated in triplicate.

Exocytosis assays. pcMSCs were seeded in 10 separate dishes, grown to confluence, and labeled with HSA-coated FNDs at 100 μg/mL for 4 h. The FND-labeled pcMSCs were then thoroughly washed by phosphate-buffered saline (PBS) to remove free FNDs and incubated

in a humidified chamber with 5% CO_2/air mixture at 37 °C. After incubation in the culture medium for a specific time period (1 – 10 days), the cells were thoroughly washed by PBS to remove exocytosed FNDs, detached by trypsinization, and sonicated in water for 1 h to break up cell membrane. The total amounts of FNDs in the suspension were finally measured by magnetically modulated fluorescence.

Table S1. Quantification of pcMSCs in the lung tissues of a xenotransplanted miniature pig by the PCR assays[a]

Samples	Wet weights (mg)	Threshold cycles (Ct)[b]	Amounts of human DNA (pg)	Numbers of pcMSCs	Numbers of pcMSCs per lung	Percentages of pcMSCs in lungs (%)
#1	25	30.70	3.579	2.878	1.669×10^4	0.072
#2	25	30.59	3.696	3.186	1.848×10^4	0.080
#3	25	30.68	3.637	3.352	1.944×10^4	0.084

[a]The total number of pcMSCs injected into the pig was 2.3×10^7 cells.

[b]The control experiment consisted of pcMSCs containing a total amount of 200 ng human DNA with Ct = 14.93.

Table S2. Quantification of HSA-FNDs and HSA-FND-labeled pcMSCs in the lung tissues of 12 xenotransplanted miniature pigs by the fluorescence assays

Treatments	Animal weights (kg)	Lung wet weights (g)	Lung dry weights (g)	Lung water contents (%)	Doses	Percentages of FNDs or pcMSCs in lungs (%)
FND (24 h)	27.0	252	53.9	78.6	2.7 mg	51
	22.2	349	74.0	78.8	2.2 mg	19
	22.5	559	89.7	83.9	2.2 mg	35
FND (48 h)	21.3	202	40.0	80.2	2.1 mg	27
	22.3	188	36.7	80.4	2.2 mg	19
	25.0	329	69.0	79.0	2.5 mg	35
FND-pcMSC (24 h)	22.5	168	35.0	79.2	2.2×10^7 cells	77
	24.3	237	48.0	79.7	2.4×10^7 cells	74
	23.2	233	52.2	77.6	2.3×10^7 cells	76
FND-pcMSC (48 h)	18.7	176	36.0	79.5	1.8×10^7 cells	62
	16.3	218	42.0	80.7	1.6×10^7 cells	75
	18.6	270	48.0	82.2	1.8×10^7 cells	69

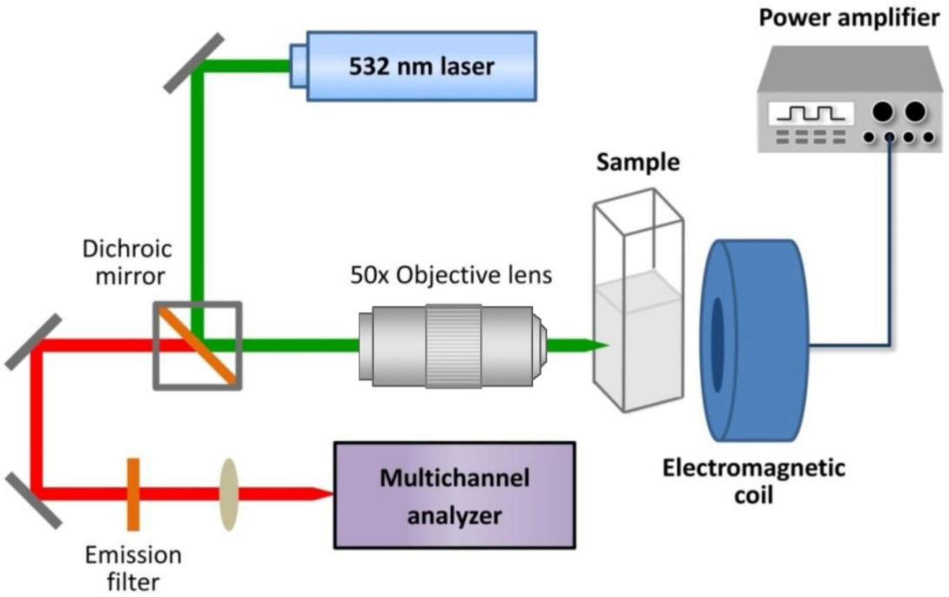

Figure S1. Experimental setup of the magnetically modulated fluorescence spectrometer for absolute quantification of FNDs in cells and tissues.

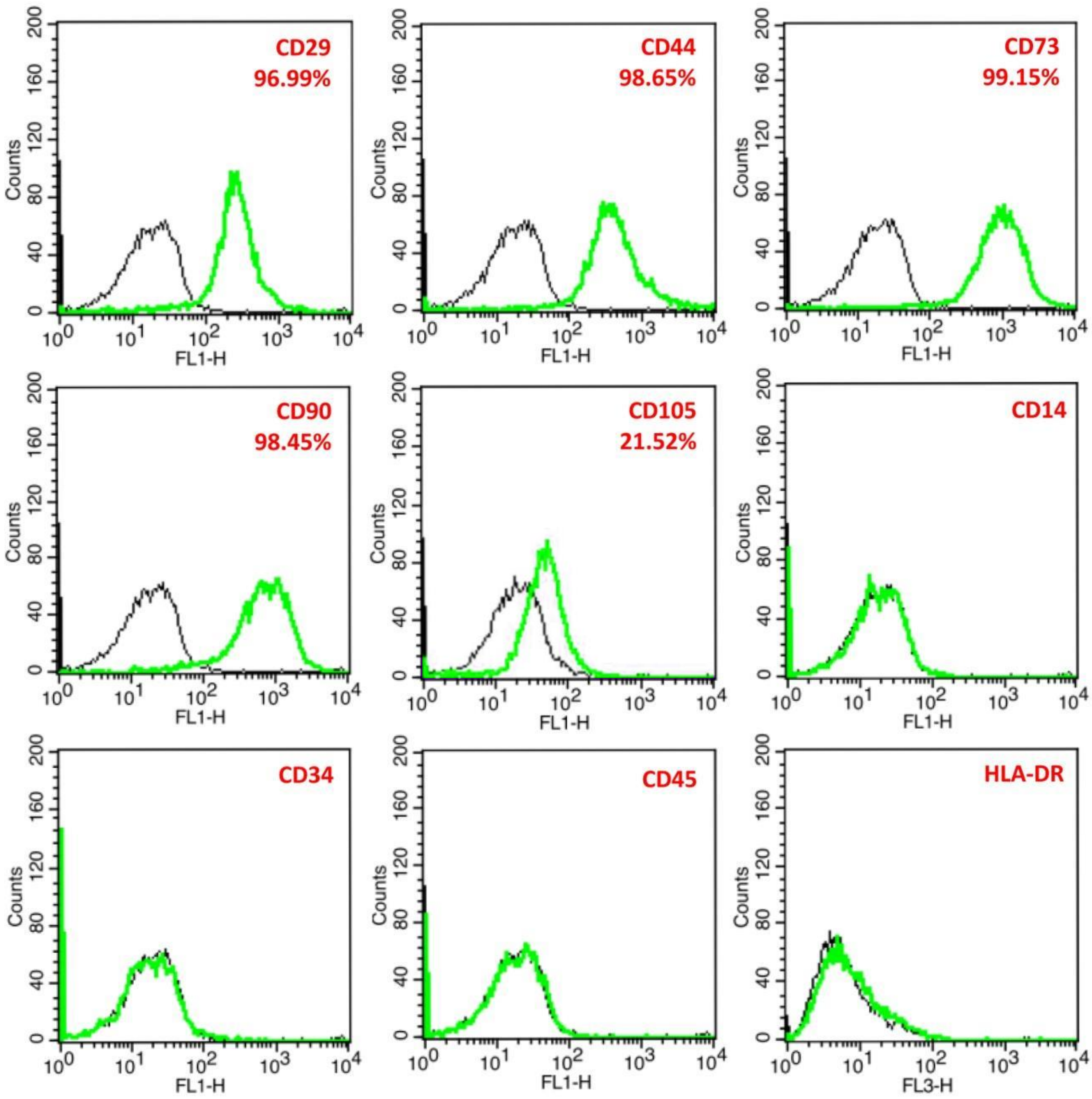

Figure S2. Immunophenotype analysis of pcMSCs, showing CD29$^+$, CD44$^+$, CD73$^+$, CD90$^+$, CD105$^+$, CD14$^–$, CD34$^–$, CD45$^–$, and HLA-DR$^–$. Control groups are in black and experimental groups in green.

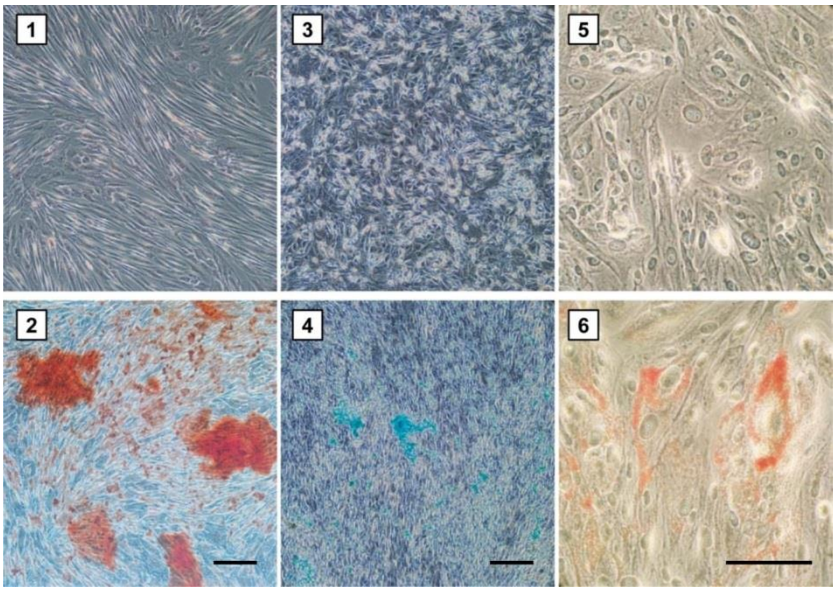

Figure S3. *In vitro* differentiation abilities of pcMSCs for osteogenesis (1, 2), chondrogenesis (3, 4), and adipogenesis (5, 6). Given in (1), (3), and (5) are results of the corresponding control groups. Scale bar: 200 μm.

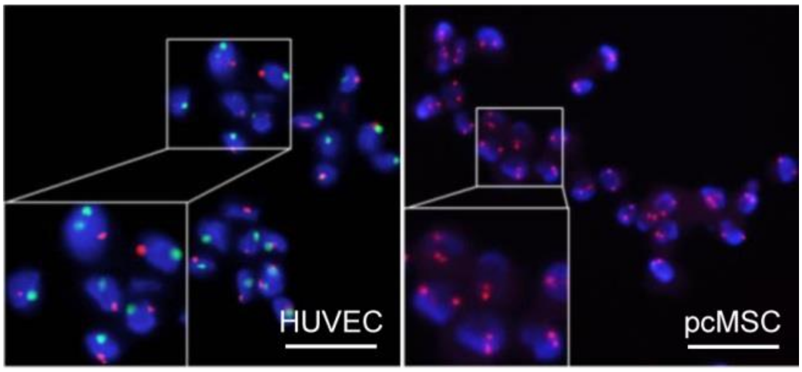

Figure S4. FISH analysis of pcMSCs. X and Y chromosomes are shown as red and green signals, respectively, and cell nuclei are stained with DAPI in blue. Human umbilical vein endothelial cells (HUVECs) served as a control, showing double-positive in both X and Y chromosomes, whereas the pcMSCs have only X chromosome in the same nuclei without Y chromosomes. Given at the left-bottom corners are enlarged views of the images in the white boxes of the individual panels. Scale bar: 50 μm.

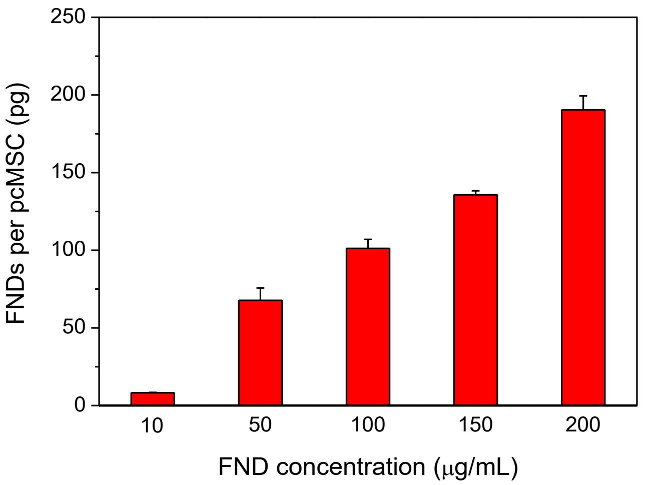

Figure S5. Amounts of HSA-FNDs taken up by pcMSCs at increasing particle concentrations, determined by MMF. Experiments were repeated in triplicate and error bars represent one standard deviation of uncertainty.

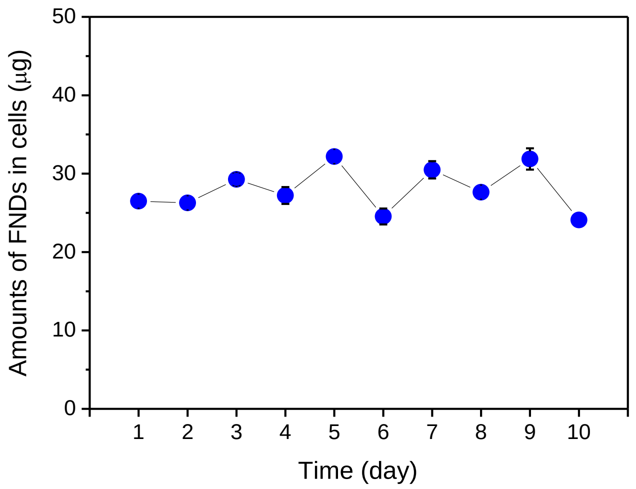

Figure S6. Variations of the total amounts of FNDs in pcMSCs cultured at 37 °C over 10 days. No significant exocytosis of FNDs was found. Experiments were repeated in triplicate and error bars represent one standard deviation of uncertainty.

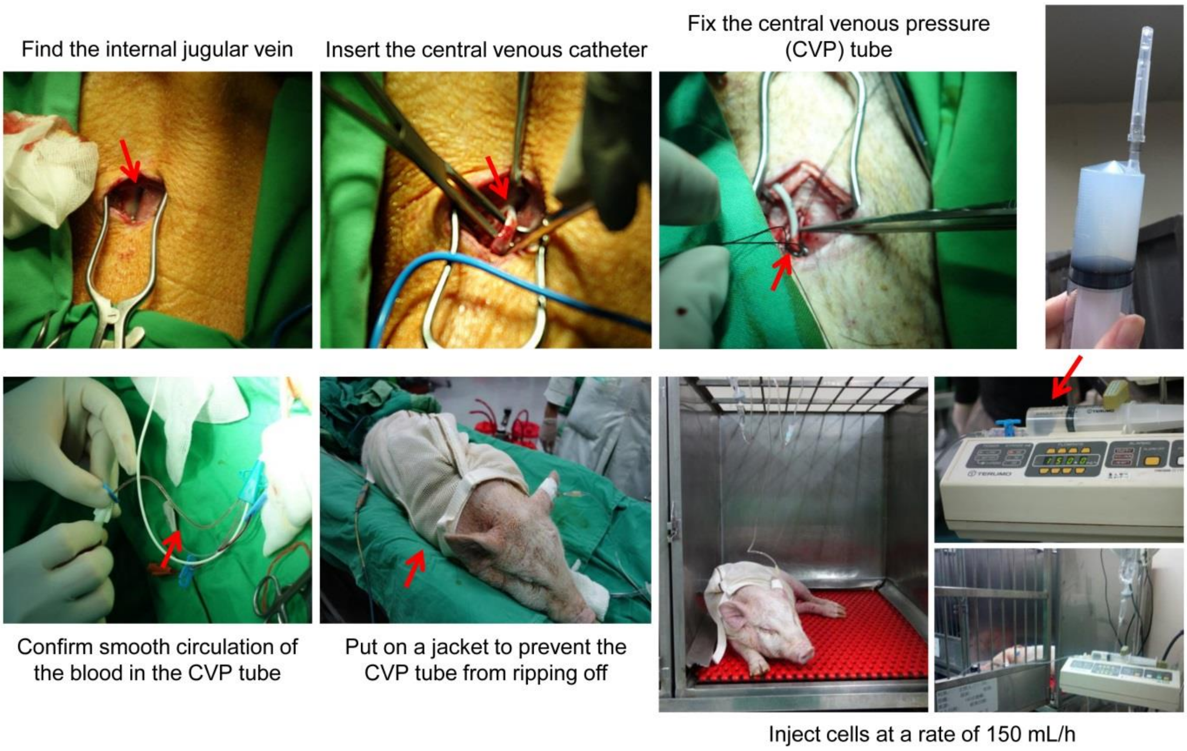

Figure S7. Central venous catheter insertion and intravenous injection of HSA-FND-labeled pcMSCs (or HSA-FNDs) into a miniature pig. A syringe pump controlled the flow of the injection at a rate of 150 mL/h.

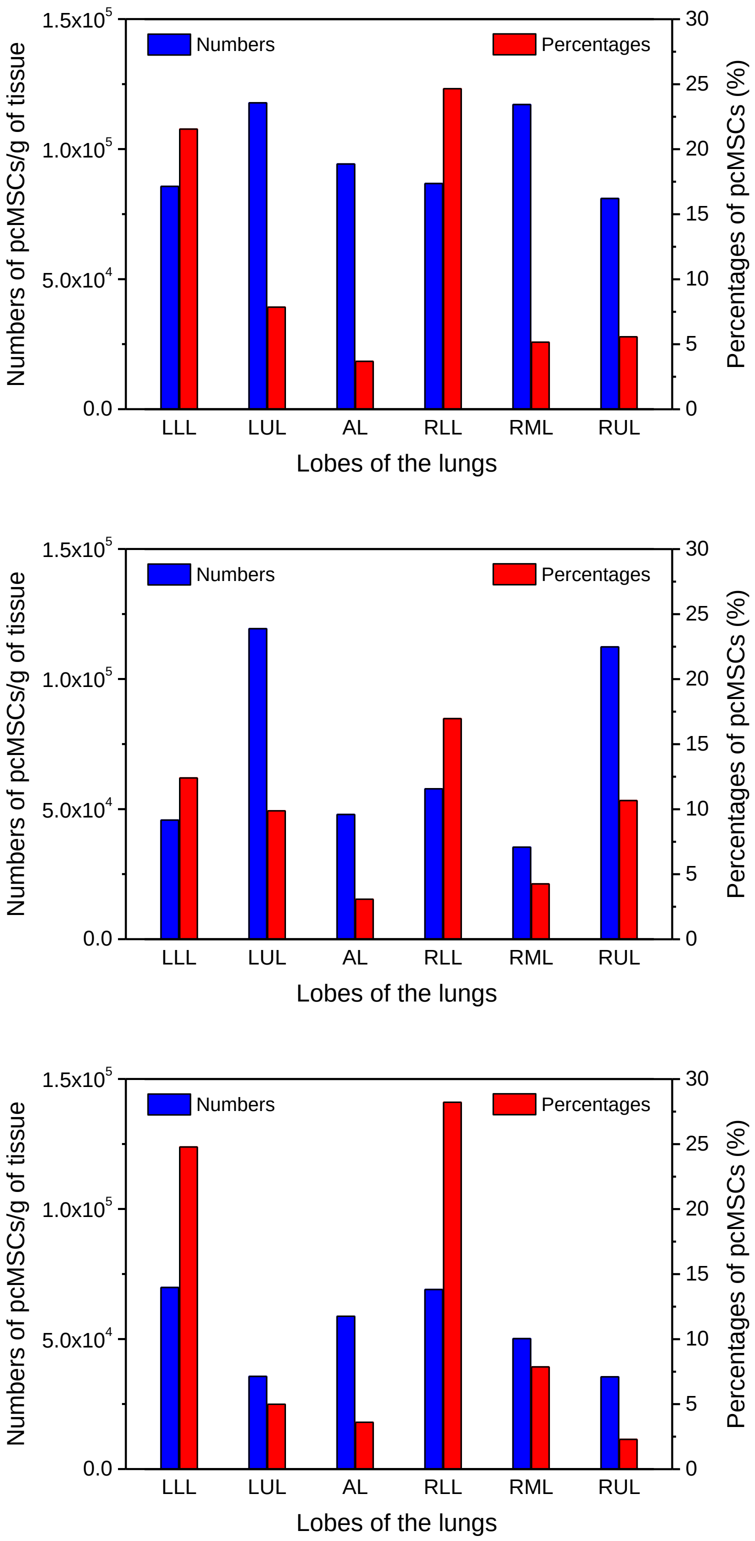

Figure S8. Distribution of HSA-FND-labeled pcMSCs in different sections of the lungs of 3 miniature pigs after intravenous injection for 48 h. LLL: left lower lobe; LUL: left upper lobe; AL: accessory lobe; RLL: right lower lobe; RML: right middle lobe; RUL: right upper lobe.

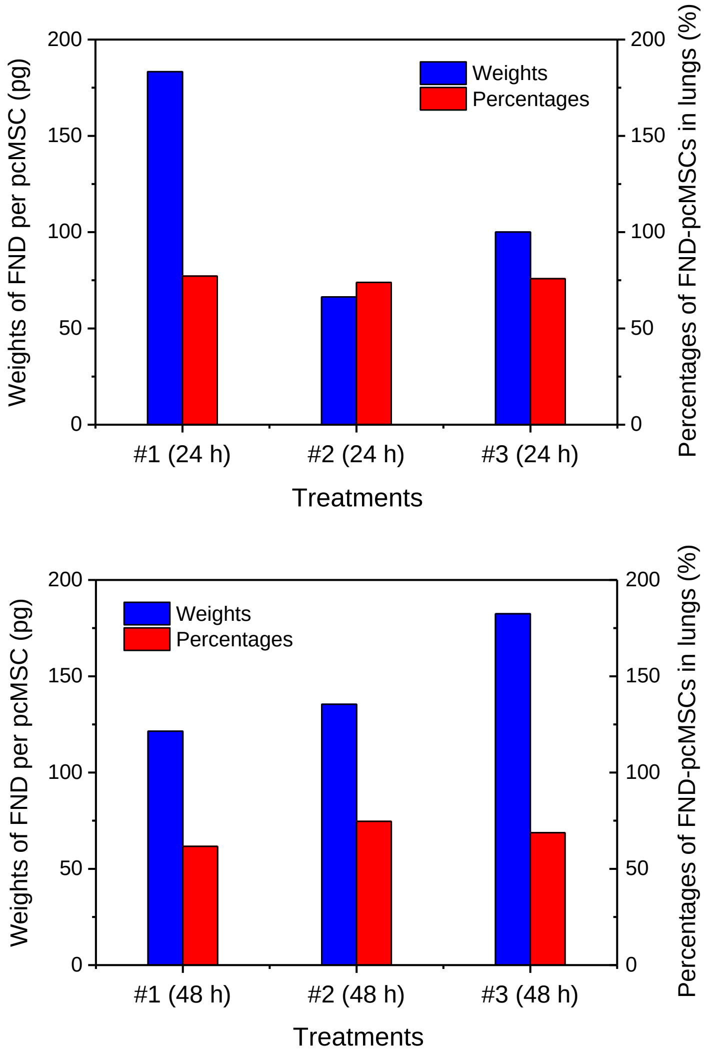

Figure S9. Amounts of HSA-FNDs taken up by pcMSCs and the corresponding percentages of HSA-FND-labeled pcMSCs found in lungs at 24 h and 48 h after intravenous injection. Each treatment was repeated in triplicate (#1 – #3). Note that the percentages of HSA-FND-labeled pcMSCs found in the lungs are independent of the amounts of HSA-FNDs taken up by the cells, showing the reliability of this quantification method. The HSA-FND concentration used for the cell labeling was 100 μg/mL.

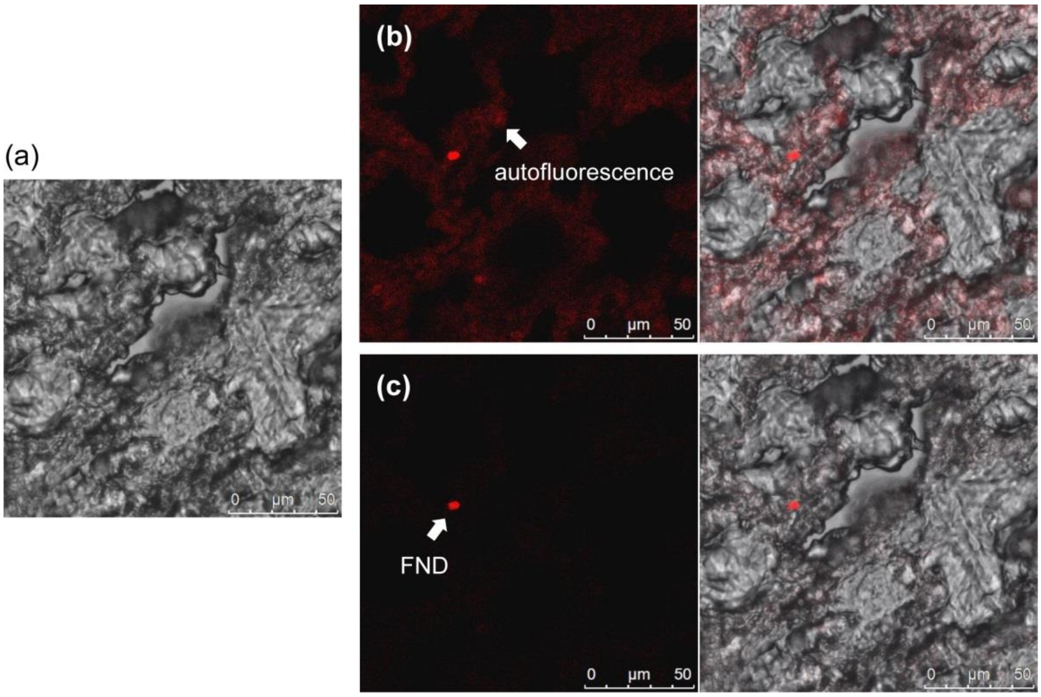

Figure S10. Fast screening of tissue sections before deparaffinization to find HSA-FND-labeled pcMSCs in the lungs of a miniature pig. (a) Bright-field image, (b) fluorescence (left) and merged bright-field/fluorescence (right) images without time gating, and (c) fluorescence (left) and merged bright-field/fluorescence (right) images with time gating at >8 ns.
